# Development of Upper Extremity Deep Vein Thrombosis in a Patient With Seronegative Myasthenia Gravis: A Case Report and Review of Literature

**DOI:** 10.7759/cureus.56086

**Published:** 2024-03-13

**Authors:** Nathan E Cohen, Andrew C Cook, Ravish Narvel

**Affiliations:** 1 Medicine, Lake Erie College of Osteopathic Medicine, Bradenton, USA; 2 Internal Medicine, Ascension St. Vincent's Riverside, Jacksonville, USA

**Keywords:** upper extremity thrombosis, seronegative myasthenia gravis, myasthenia gravis crisis, upper extremity dvt, myasthenia gravis (mg)

## Abstract

We present the case report of a patient with seronegative myasthenia gravis (MG) who was admitted for metabolic encephalopathy and acute on chronic hypoxic respiratory failure secondary to an MG crisis three days after an intravenous immunoglobulin treatment. In the intensive care unit, her MG was managed with intravenous immunoglobulin, plasmapheresis, prednisone, and pyridostigmine. During the course of her visit, she had urosepsis along with a left chest port that had cultured positive for *Pseudomonas aeruginosa* and developed a right upper extremity deep vein thrombosis (UEDVT) and superficial thrombosis in the left upper extremity despite being on heparin therapy. She had a transient drop in platelets to below 150,000 that resolved within a day. We analyzed the variables of this case report and reviewed the literature of similar cases to elucidate the factors that may have led to the development of the UEDVTs. The patient had many factors in her past medical history that could have contributed to her thrombosis including morbid obesity and prior history of pulmonary embolisms. It is hypothesized that MG disturbs the endothelial cell lining through an increased inflammatory state that could also be a causative factor. There is no definitive way we could link MG as a causative factor due to a lack of testing to assess alteration in the integrity or functionality of her endothelium. A case report we reviewed showed a presentation of UEDVT in an MG patient due to a thymoma compressing the subclavian vein. However, this is not the case in this example due to the patient having a history of thymectomy. She was also at risk due to her hospital stay which led to immobility and placement of a central venous catheter. We conclude the formation of the UEDVT was likely a combination of these factors.

## Introduction

Myasthenia gravis (MG) is an autoimmune condition in which the body produces antibodies against proteins that are responsible for the function of the neuromuscular junction. The most common protein that is targeted in this disease are acetylcholine receptors (AChR) which are responsible for binding to release acetylcholine (ACh) from the pre-neuromuscular synapse, internalizing ACh at the post-synaptic cleft and inducing a cascade of reactions that leads to muscle contraction [1]. It has been reported that 80-90% of cases of myasthenia crisis involve antibodies that target AChR. Another protein that is targeted is muscle-specific tyrosine kinase (MuSK), a protein responsible for clustering AChR in an effort to generate a stronger signal for muscle contraction. Clustering of AChR is first induced by a nerve proteoglycan called agrin which binds to lipoprotein-related protein 4, activating MuSK. MuSK then activates DOK7, a cytoplasmic adaptor protein that recruits AChR binding proteins including rapsyn that ultimately aggregate AChR [2]. MG caused by MuSK antibodies has a prevalence of 40-70% among patients in Europe and Japan who are AChR seronegative [3]. Knowing the physiology of how the neuromuscular junction works can bring an understanding that antibodies against any of the proteins responsible for functioning the neuromuscular junction can lead to the development and signs of MG. The abovementioned causes of MG are deemed seropositive causes of MG because they can be detected in assays that seek these specific antibodies in blood/serum. 

Another class of MG is seronegative MG because neither AChR nor MuSK antibodies are not detectable. Some cases of seronegative MG can be due to low sensitivity assays as more than 50% of seronegative MG actually have detectable AChR antibodies but these antibodies are of low affinity [4]. Other proteins that have been found to be targets of seronegative MG include low-density lipoprotein receptor-related protein 4, actin, myosin, rapsyn, and titin to name a few, all involved in the neuromuscular junction [5,6]. 

The prevalence of MG has increased over time partly due to better technology in recognition of disease markers including AChR antibodies along with better medication and management. MG has a bimodal distribution with females predominantly affected under age 40 with a 7:3 ratio, while males are predominantly affected in those over age 50 with a 3:2 ratio [7]. MG is slightly more prevalent among African Americans, and mortality rates tend to be higher in males than females [8]. Seronegative MG represents a minority of cases with a prevalence of 10-15% [9]. 

This seronegative MG patient developed a right upper extremity deep vein thrombosis (UEDVT) along with superficial thrombosis of the left arm. Thrombosis is classically due to three factors known as Virchow's triad, endothelial lining dysfunction, blood flow disruption, and hyper-coagulable states [10]. UEDVTs are a more rare subset comprising 10% of all DVTs [11]. The highest risk factors for UEDVTs specifically are central venous catheters, malignancies, underlying diseases, immobility, and surgical interventions [12]. Many of these factors are present in this case and are further examined in the following sections.

## Case presentation

A 69-year-old female with a past medical history of chronic obstructive pulmonary disease (COPD), congestive heart failure (CHF), diabetes mellitus (DM), gastroesophageal reflux disease (GERD), hyperlipidemia (HLD), morbid obesity, and seronegative MG was admitted to the hospital from a skilled nursing facility for altered mental status, acute on chronic respiratory failure secondary to an MG crisis, urinary tract infection (UTI) complicated by *Klebsiella pneumoniae* and *Proteus mirabilis*, as well as a left chest port that cultured positive for *Pseudomonas aeruginosa*. She was intubated for 10 days with stable vitals and a normal arterial blood gas (ABG) that was drawn on the day of her extubation. Her MG has failed multiple disease-modifying therapies including Rituxan, Imuran, CellCept, and Cytoxan.

The patient was also on vasopressor support for 10 days with norepinephrine. After extubation, she was put on bilevel positive airway pressure (BiPaP) at night and a 3 L nasal cannula during the day and was transferred to the floor unit from the intensive care unit (ICU) two days after her extubation. During her admission to the ICU, she had five plasmapheresis (PLEX) sessions on alternate days that spanned over a period of nine days, and her prednisone was increased from 15 mg daily to 40 mg daily two days after admission for 10 days which was then tapered down to 20 mg for one week. She was further tapered to 15 mg daily after her one-week regimen of 20 mg prednisone and is currently on this dose. Her pyridostigmine regimen of 120 mg four times a day of two tablets remained the same throughout her hospital course. The patient had two therapy sessions on two consecutive days of 40 g intravenous immunoglobulin (IVIG) and is scheduled to receive IVIG every three weeks. Physical therapy/occupational therapy/speech therapy (PT/OT/ST) have been followed during her admission, and she was discharged back to the skilled nursing facility for rehab along with PT/OT. She has a history of recurrent UTIs and kidney stones.

The patient's DM was managed by the Diabetes Management Services (DMS) team, and her metformin was discontinued due to elevated liver enzymes with an aspartate aminotransferase (AST) of 535 and alanine transaminase (ALT) of 272 on the day of admission. Her liver enzymes eventually decreased within normal values by admission day 6. The patient was started on a sliding scale insulin with adjustments according to blood glucose values daily. She had anemia throughout admission, and her hemoglobin (Hgb) and hematocrit (Hct) were monitored daily. She had a blood transfusion on day 8 of admission for a Hgb of 6.8 for which her Hgb stayed stable above 7.0 throughout her admission. The cardiology team had been following up due to heart failure with a preserved ejection fraction of 55-60% as of her last echocardiogram seven months prior. The patient also has a history of premature atrial contractions and premature ventricular contractions. She also started to develop diffuse skin sloughing on her back, mammary folds, and groin area along with erythema and tenderness on day 17 of her admission. 

For the patient's urosepsis, she had received a single dose of Rocephin 1 g IV on her day of admission and then a five-day course of cefepime 1 g IV every six hours. The patient also received a single dose of vancomycin 2000 mg IV on her second day of admission followed by three days of vancomycin 1500 mg IV and then enteral vancomycin 125 mg for 10 days for *Clostridioides difficile* (*C. diff*) prophylaxis with which she has a recurrent history of. She had resolution of her symptoms until hospital day 14 where she was complaining of supra-pubic tenderness. A urine culture was obtained, positive for *Proteus* and gram-negative rod UTI, for which she was started on cefepime 1 g IV every six hours for a total of five doses for two consecutive days followed by cefdinir 300 mg twice a day for six days. She was also started on a six-week tapering schedule of oral vancomycin 125 mg due to recurrent *C. diff* colitis with which she started developing painful diarrhea on the second day of cefepime and was put on loperamide as needed; by this point in time, she reported relief of supra-pubic tenderness. 

**Figure 1:**
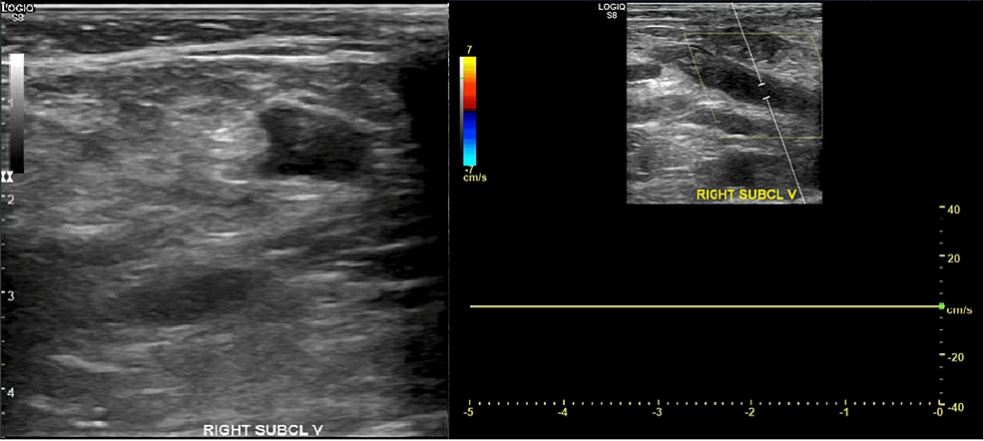
Ultrasound of the right subclavian vein DVT The left image reveals a non-compressible right subclavian vein. The right image reveals a lack of blood flow through the right subclavian vein where the thrombus was located. DVT: deep vein thrombosis

She had also developed a right UEDVT and superficial thrombosis of the left arm recognized on the ninth day of admission through ultrasound (US). The findings included occlusive thrombus within the right subclavian, basilic, and cephalic veins, non-occlusive thrombus within the right axillary vein, and occlusive thrombus within the left cephalic vein despite being on a heparin drip of 5,000 units every eight hours for nine days starting on the second day of admission. Her platelet count at the time was 147,000 but remained stable above 150,000 throughout the rest of her admission. She has a past medical history of recurrent pulmonary embolisms (PEs) that were managed with an inferior vena cava (IVC) filter. A coagulation panel was performed on the day her UEDVTs were found, revealing an elevated protime of 14.5 and an international normalized ratio (INR) of 1.5 with a fibrinogen level of 192 and prothrombin time of 31.9. She was treated with seven days of Eliquis 10 mg loading dose followed by 5 mg daily for three months. She was discharged after 20 days of being admitted to a long-term rehabilitation facility. Figure 1 is the US image of the UEDVT.

## Discussion

Here, we present the case of a patient with a history of seronegative MG that later developed right UEDVT and superficial thrombosis of the left upper extremity. We have examined the literature and have found only one study which reported such a case. In this example, it was determined that the patient's thymoma led to external compression of the innominate vein [13]. This disruption of venous flow led to the DVT. This would not be possible in our case due to the patient having a thymectomy. In a different study, Lin et al. reported a case where the patient's MG led to a PE [14]. They hypothesized that endothelial cell injury was due to increased inflammatory mediators from the patient's MG. Unfortunately, due to a lack of testing of the integrity and functionality of her endothelium, we cannot definitively say that her MG was a possible factor for her thrombosis.

Thrombosis is caused by three factors known as Virchow's triad which includes endothelial damage, alteration of blood flow such as stasis, or a hyper-coagulable state [10]. The first factor which may have contributed to the patient's DVTs was her prolonged immobility leading to stasis. The patient was hospitalized and had limited mobility for 19 days. Development of her thrombosis could be attributed to a number of factors including her central line catheter placement which is believed to be a primary factor as well as possible inflammatory changes hypothesized to be associated with MG [15]. Hyper-coagulable states must also be assessed to determine the risk factors leading to this patient's DVTs. She does not have any known coagulation genetic mutation diseases or underlying malignancies that would contribute to her DVT. Heparin-induced thrombocytopenia (HIT) as a potential cause of a hyper-coagulable state was investigated since the patient was on heparin for eight days before the DVTs developed. Her platelets upon heparin initiation were 277k and 146k two days later, causing an estimated 47% decrease. Using the 4T's prediction test, our patient scored a 5 which indicates an intermediate probability of type 2 HIT. However, the drop in platelets was within the first two days of administration suggesting type 1 HIT, which is not associated with a hyper-coagulable state. The patient was not tested for platelet factor 4 (PF4) heparin immunoassay or functional platelet activation assay, so an ultimate determination cannot be made. Additionally, the patient received multiple rounds of PLEX for her MG during the stay. So, in theory, this would have removed any potential IgG antibodies against heparin/PF4 immunocomplex, making the diagnosis of type 2 HIT less likely than type 1. 

The patient has risk factors in her medical history which predispose her to an increased risk of thrombosis. She has a history of PEs with an IVC filter. Additionally, her advanced age and morbid obesity also increase her risk of thrombosis. A combination of all these variables in her past medical history and conditions with her hospital stay all contributed to the formation of her upper extremity thrombosis. Further research is needed in regard to MG and its association with inflammatory states and how these factors could be correlated in creating an environment favoring thrombosis formation.

## Conclusions

We investigated the case of a patient with a history of seronegative MG who later developed a right UEDVT and superficial thrombosis of the left upper extremity. Many factors likely contributed to the development of the DVTs. The patient's past medical history of PEs, morbid obesity, and age in addition to the theoretical possibility of inflammation associated with her MG all likely played a role in her recurrent history of thrombosis.
